# V-Notes Sentinel Lymph Node Staging for Endometrial Cancer: A Systematic Review

**DOI:** 10.3390/jcm14186451

**Published:** 2025-09-12

**Authors:** Mihai Nădăban, Oana Balint, Cristina Secoșan, Alexandru Marius Furău, Flavius Olaru, Laurențiu Pirtea

**Affiliations:** 1 Doctoral School, University of Medicine and Pharmacy “Victor Babes”, 300041 Timisoara, Romania; mihai.nadaban@umft.ro; 2 Obstetrics-Gynecology I Department, University of Medicine and Pharmacy “Victor Babes”, 300041 Timisoara, Romania; secosan.cristina@umft.ro (C.S.); olaru.flavius@umft.ro (F.O.); pirtea.laurentiu@umft.ro (L.P.); 3 Department of Oncology, “Vasile Goldis” Western University of Arad, 310414 Arad, Romania; furau.marius@uvvg.ro

**Keywords:** V-NOTES surgery, sentinel lymph node, endometrial cancer, minimally invasive surgery

## Abstract

**Background/Objectives**: Sentinel lymph node (SLN) mapping has become a standard approach in the surgical staging of early-stage endometrial cancer, aiming to reduce the morbidity associated with full lymphadenectomy while preserving diagnostic accuracy. Vaginal Natural Orifice Transluminal Endoscopic Surgery (V-NOTES) represents a novel, minimally invasive approach for SLN mapping that may offer specific benefits in terms of access, cosmetics, and recovery, particularly in select patient populations. **Methods**: A comprehensive literature search was conducted in the main databases for studies evaluating the use of V-NOTES for sentinel lymph node mapping in patients with endometrial cancer. **Results**: Of the 12 included studies, three were observational cohort studies, while the remaining were case reports and series. The mean patient age was 59.6 years, with a majority being overweight or obese. V-NOTES was performed using both transperitoneal and retroperitoneal approaches, with indocyanine green (ICG) being the most used tracer. The overall mean SLN detection rate was 98.19%, with a bilateral detection rate of 93.7%. The average operative time was 155 min, the mean blood loss was 72.03 mL, and the average hospital stay was 2.4 days. The complication rate was low (3.9%), with no conversions to laparotomy reported. **Conclusions**: V-NOTES appears to be a feasible and safe alternative for SLN mapping in early-stage endometrial cancer, with high detection rates and favourable perioperative outcomes. However, due to the limited number of comparative studies and small sample sizes, further prospective research is needed to establish its efficacy relative to traditional minimally invasive techniques and to determine the optimal patient selection criteria.

## 1. Introduction

Endometrial cancer ranks among the most prevalent malignancies in developed nations. Its rising incidence is primarily attributed to the concomitant increase in the prevalence of one of its most significant risk factors: Obesity [1]. Surgical staging of endometrial cancer is a crucial step in determining the extent of disease spread and guiding treatment. Together with the currently recommended preoperative work-up, it provides information about tumour grade, depth of myometrial invasion, and lymphatic involvement [1].

Lymph node evaluation is a crucial component of the staging process, providing valuable insights into the necessity of adjuvant treatment and the patient’s prognosis [2]. Traditionally, systematic lymphadenectomy includes the dissection of pelvic and para-aortic nodes extending up to the renal veins. A series of extensive retrospective studies have correlated systematic lymphadenectomy with extended survival, particularly in high-risk subtypes of endometrial cancer [3]. However, systematic lymphadenectomy was also associated with increased risks for the patients and increased morbidity rates [4]. Their incidence varies in the literature, but it has been observed that a significant proportion of patients undergoing such a procedure present direct complications such as chronic lymphedema, lymphocysts, infections, or vascular and neurological lesions [5].

The sentinel node is defined as the first node involved in the drainage of the primary tumour to the regional lymph basin. To map it, a tracer is injected into the tumour area. It will migrate through the lymphatic vessels, initially reaching the sentinel node, which will be identified and surgically removed. If metastatic cells are not identified in this node, it can be assumed that the other non-sentinel nodes will also not contain metastatic cells [6]. As this concept became the standard for the surgical management of breast cancer and melanoma, the technique was also applied to other types of cancer, such as endometrial cancer. Although research in this field on gynecologic cancers was limited to a small number of cases for many years, the sentinel node technique has gained popularity recently due to the standardisation of the method [7,8]. The risk of lymph node metastasis in endometrial cancer varies between <5% and 40% depending on tumour grading, tumour histology, and myometrial invasion. Current recommendations of professional association guidelines regarding indications for sentinel node evaluation are: it may be considered for staging purposes in patients with low- or intermediate-risk disease and should be performed in patients with intermediate–high- or high-risk disease [9].

V-NOTES or Vaginal Natural Orifice Transluminal Endoscopic Surgery is an emerging minimally invasive surgical technique that uses a natural orifice, in this case, the vagina, for surgical access. This surgical approach has been associated with reduced blood loss, shorter operative time, shorter hospitalisation time and a lower rate of postoperative morbidity as compared with classical laparoscopy [7]. In recent years, numerous publications have reported the use of the V-NOTES technique for different types of gynecologic procedures [2]. Several systematic reviews and meta-analyses found that V-NOTES is a feasible technique for hysterectomy and other benign gynecologic pathology [3,4]. Additionally, it has emerged as a superior technique for obese patients, as the conventional approach can prove to be challenging [5]. V-NOTES was applied for the first time for sentinel node mapping in endometrial cancer in 2016 by LeBlanc, and a few years later, Baekelandt described a new retroperitoneal approach [6,8]. The literature concerning this topic is increasing, but mainly by low-quality papers such as case reports or case series. No randomised controlled trial of V-NOTES in endometrial cancer or any related topic has been published.

This review presents a comprehensive summary of existing studies conducted on the V-NOTES technique employed for sentinel node mapping in endometrial cancer patients.

## 2. Materials and Methods

The present review aims to present the existing evidence regarding the use of V-NOTES techniques for sentinel node mapping in endometrial cancer patients.

- **Eligibility Criteria**

1. Study design: Original articles including case studies, observational studies and randomised trials.
2. Method: Studies that involved performing the sentinel node mapping using the V-NOTES technique.
3. Human involvement.
4. Language: Studies published in English.
5. Full text availability.

- **Information Sources and Search Strategy**

**Figure 1:**
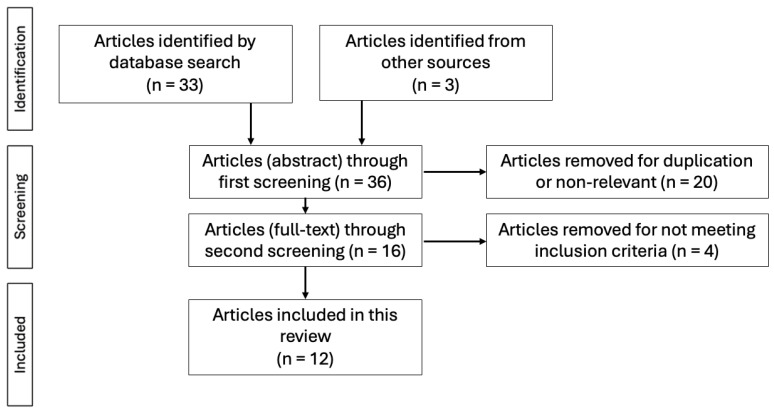
PRISMA chart.

The search for eligible studies was performed in medical databases such as PubMed, Scopus and Web of Science. Studies published between January 2016 (the year of the first report) and April 2025 were considered. The search strategy used multiple associations of keywords: “V-NOTES”, “endometrial cancer”, “sentinel lymph node” and “sentinel node mapping”. Additionally, studies matching including criteria identified in the references of the studies found by the search strategy were included manually. This systematic review was conducted in accordance with the Preferred Reporting Items for Systematic Reviews and Meta-Analyses (PRISMA) guidelines. A completed PRISMA checklist is provided in Figure 1 to ensure transparency.

- **Study Selection**

After the initial search, 35 articles were found. For each result, titles and abstracts were screened for eligibility. Articles that did not meet the inclusion criteria, were not relevant to the review’s purpose, or were duplicate studies, were excluded. In the next phase, the full text of the remaining 15 studies was reviewed, and non-relevant articles were also eliminated. We mention that 2 of the articles, which were relevant for our review, were available only as video articles and were excluded. Twelve (*n* = 12) articles were ultimately included in the review.

- **Data Extraction**

Data relevant to the study objectives including first author, year of publication, design of the study, number of the patients included, pre-operative patient’s characteristics, operative parameters and outcomes of the SLN mapping including mean number of lymph nodes detected and detection rates were extracted, and a database was created in Jamovi software ver. 2.6.24 (https://www.jamovi.org, accessed on 15 April 2025).

## 3. Results

In total, a number of 12 articles were considered eligible to be included in the current review, from an initial search of 36 articles.

The search strategy for this review is presented in Figure 1.

### 3.1. Characteristics of the Studies Included

Studies published between 2016, the year of the first report of the V-NOTES technique in SLN mapping, and 2025 were included in this review. As this analysis is focusing on an emerging procedure, we decided to include all study designs, including case reports and case series. Only three of the twelve studies were observational cohort studies where the V-NOTES technique was compared with other procedures, while the other nine studies were case reports (*n* = 2) or case series (*n* = 7). The studies included in this review were assessed for the risk of bias using the Newcastle-Ottawa Scale for cohort studies and JBI’s critical appraisal tools for case reports and case studies [9,10]. Overall, the majority of studies demonstrated either moderate or high risk of bias, primarily due to limitations in selection methods, inadequate control for confounding variables, and incomplete or short follow-up periods. These methodological concerns may affect the reliability of the reported outcomes and should be considered when interpreting the findings.

### 3.2. Characteristics of the Population Included

The twelve eligible studies included a total of 253 patients. The case report studies included between 4 and 64 patients, while the cohort studies included between 19 and 54 patients. The mean age of the population recruited in the studies was 59.6 years.

Several patient characteristics were observed across nearly all the included studies. Except for a single case report, body mass index (BMI) was reported in all studies, ranging between 24.2 kg/m^2^ and 31 kg/m^2^, with a mean of 27.21 kg/m^2^. The percentage of patients with a Body Mass Index (BMI) exceeding 25 kg/m^2^ (categorised as overweight or obese) included in the population for the studies was reported in eight of the twelve articles, ranging between 26% and 100%. Three studies included only overweight and obese patients. Another important factor for vaginal surgeries is the parity of the patient. Eight of the twelve studies reported the mean parity of the population included, which ranged between 1.8 and 3.2 births. The final significant patient characteristic included in most of the studies was the percentage of patients who had prior abdominal surgery, reported in ten of the twelve studies. This variable ranged from 25% to 100%, with a mean of 48.7%. Three of twelve studies had as exclusion criteria for their study population extensive adhesiogenic conditions. Patient’s characteristics are presented in Table 1.

### 3.3. Histological and Imaging Characteristics

Four variables were observed in most of the studies. These were pre-operative variables. Histology of biopsy specimens was mentioned in nine of the twelve studies. Six studies included only patients with endometrioid carcinoma, two studies included endometrioid carcinoma and complex atypical hyperplasia, and one study included endometrial tumours of all histological types and complex atypical hyperplasia. Grading of the tumour was available for the same nine studies. Eight of those nine studies included tumours of low grade (G1 and G2), and one study also included high-grade (G3) tumours.

In nine of the twelve studies, preoperative imaging information (ultrasound and/or MRI) was available. The details provided varied, ranging from a general mention of the procedure being performed to a detailed description of specific findings, such as tumour dimensions, localisation, and absence of lymphatic or distant metastases.

Staging was available in ten studies, either explicitly mentioned or indirectly inferred by providing sufficient data to determine the stage of tumours. Consequently, nine studies exclusively included patients with stage I tumours, whereas the study that also involved G3 tumours included patients with stage II tumours, specifically stage IIA. Histological and imaging characteristics are presented in Table 2.

### 3.4. Surgical Outcomes

All included patients underwent a total hysterectomy with bilateral salpingo-oophorectomy as the primary procedure. SLN mapping was performed using the V-NOTES technique by transperitoneal approach in five cases and the newer retroperitoneal approach in eight cases. Ten of the twelve studies utilised indocyanine green (ICG) as the tracer, while one study combined ICG with carbon nanoparticles (CNPs), and another utilised only CNPs. The laparoscopic conversion rate varied between 0% and 14%, and no conversion to laparotomy was necessary in any case. The mean operation time available for eleven studies was 155 min. In ten of the twelve studies, the mean blood loss was mentioned, and it was an average of 72.03 millilitres. Four studies reported the difference between the pre-operative and post-operative haemoglobin value, with a mean of minus 1.3 g per decilitre. No blood transfusion was necessary in any of the studies included. Post-operative hospital stay was available in ten studies, with a mean of 2.4 days. Surgical outcomes are presented in Table 3.

### 3.5. SLN Mapping Outcomes

The mean success rate was 96.4%, with six studies reporting a success rate of 100%. The mean total lymph nodes identified was reported in nine studies and ranged between 2 and 12.5 lymph nodes. Mean right and left lymph nodes were reported in seven studies and ranged between 1 and 5.7 nodes for the right side and 1 to 6.2 nodes for the left side. Overall detection rate was available for ten of the twelve studies and had a mean of 98.19%. Bilateral detection rate had a mean of 93.7% and unilateral detection rate had a mean of 6.7%. When comparing detection rate based on the SLN mapping approach, no significant difference is observed (*p* = 0.67). The distribution of the SLN by anatomic location was available in only six studies: external iliac 10.5–77.3%, obturator 57.3–84%, internal iliac 2–4.8%, common iliac 2.9–22.7% and presacral 0.3–0.9%. SLN mapping outcomes are presented in Table 4.

### 3.6. Complications and Follow-Up

Complications during surgical procedures were reported in eleven of the twelve studies. Complications were encountered in four studies, involving ten patients out of the 253 patients included in all the studies (3.9%). The most frequent complications were vascular injuries with active bleeding or hematoma formation in six patients, followed by bladder injuries in four patients.

## 4. Discussion

This review aimed to assess the current evidence regarding the use of the vaginal natural orifice transluminal endoscopic surgery (V-NOTES) technique for sentinel lymph node (SLN) mapping in endometrial cancer staging. As a relatively novel approach, V-NOTES is gaining traction for its minimally invasive nature and potential benefits in gynecologic oncology. The inclusion of diverse study designs, including case reports and case series, reflects the emergent nature of this technique and the still limited but growing body of literature. Thus, the result of this review is associated with a high risk of bias, necessitating careful interpretation of the results.

Our review included 12 studies with a total of 253 patients, demonstrating that although the overall sample size remains modest, early clinical experiences with V-NOTES in SLN staging are increasingly reported. The patients’ characteristics that were encountered in almost all the studies included highlight the applicability of V-NOTES in real-world settings, including in populations often considered technically challenging for traditional laparoscopy. Obesity is a well-established risk factor for endometrial cancer, accounting for a substantial proportion of patients who necessitate surgical intervention for this disease [23]. The increased abdominal wall thickness in these individuals can complicate conventional open or laparoscopic surgical procedures, thereby elevating the likelihood of surgical complications and adverse outcomes. Furthermore, most of these obese patients also exhibit multiple comorbidities that will increase the anaesthesia risks, necessitating shorter operative times. The V-NOTES technique represents an alternative for these individuals [24]. In our review, the mean body mass index (BMI) of the patients included was 27.21 kg/m^2^, with overweight and obese patients comprising 26 to 100% of the study population. A notable advantage of the V-NOTES technique over abdominal surgery is its suitability for patients with prior abdominal surgeries and the potential for extensive peritoneal adhesions, as it avoids the need to pass through the abdominal wall. Although some highly adhesiogenic conditions or procedures, such as rectovaginal endometriosis or pelvic radiotherapy, have been considered contraindications to V-NOTES in a recent expert consensus, procedures like prior caesarean section, total hysterectomy, or sacrocolpopexy did not reach a general agreement [25]. In our review, the history of abdominal surgery was a pre-operative variable in ten of the twelve studies and ranged between 25 and 100% of the population included.

The most frequent histological characteristics encountered in the studies included endometrioid carcinoma type, low-grade (G1 and G2) tumours and patients with stage I disease. The predominance of these characteristics of endometrial cancers in the studies is consistent with current guidelines recommending sentinel node staging primarily in low- to intermediate-risk cases [26].

The surgical characteristics demonstrate encouraging operative outcomes and highlight the reported advantages of the V-NOTES technique. The mean operation time (155 min) and low blood loss (72.03 mL), coupled with a low rate of conversion to laparoscopy (up to 14%) and no conversions to laparotomy, suggest that V-NOTES can be performed safely in selected patients. Also, the average hospital stay of 2.4 days is consistent with the benefits expected from minimally invasive approaches. The effectiveness and safety of the V-NOTES procedure in benign gynecologic surgery have been reported in numerous studies. In a recent systematic review and meta-analysis, V-NOTES had significantly lower operative times, shorter hospital stay, post-operative pain scores and similar estimated blood loss, haemoglobin change on day 1 compared with conventional laparoscopy [3]. Data concerning V-NOTES in gynecologic malignancy is poor. Most reports involve endometrial cancer, and only a few case reports on its use in ovarian and cervical cancer [27,28,29].

While all the patients included in the review had total hysterectomy with lateral salpingo-oophorectomy as the primary procedure, the SLN mapping was performed either through a transperitoneal approach or through a retroperitoneal approach. These techniques differ in surgical access, anatomical exposure, and procedural complexity. The transperitoneal approach enters the peritoneal cavity directly through a posterior colpotomy, offering a safety and efficacy comparable with conventional laparoscopy [22]. Its disadvantages are the need for bowel mobilisation, a more extensive dissection of peritoneal surfaces and suboptimal visualisation of the caudal part. In comparison, the retroperitoneal approach, first described by Baekeland in 2019, creates a retroperitoneal space without opening the peritoneal cavity, providing a better visualisation of the lymphatic system more logically in a caudal to cranial direction. Its main disadvantage is the higher technical skill required [8]. In our review, the retroperitoneal approach was the most used (eight studies). A comparison summarising the main characteristics of the retroperitoneal versus transperitoneal approaches in the V-NOTES procedure is presented in Table 5.

Of particular note, we mention the high detection rates achieved through V-NOTES. A mean overall SLN detection rate of 98.19%, with bilateral detection in 93.7% of cases, compares favourably to traditional laparoscopic or robotic techniques. For SLN mapping by conventional laparoscopy and robotic laparoscopy, an overall detection rate of 62% to 96% is reported. However, the bilateral detection rate is lower, with 34% to 88% [30]. In our review, we emphasise that the highest detection rates, reaching 100%, were observed in studies involving one or fewer than ten patients. Consequently, these rates should be interpreted with caution. This observation may contribute to the overall detection rate observed. The results indicate that, with experienced practitioners, V-NOTES can effectively achieve oncologically appropriate nodal staging. Several studies have investigated the learning curve for V-NOTES, demonstrating an initial competency of 12 cases of hysterectomy or 20 cases of ovarian cystectomy, and reaching mastery after 53 cases of hysterectomy [31,32]. However, to our knowledge, no studies have been published that explore the learning curve associated with V-NOTES SLN mapping. Although one of the most important advantages of the V-NOTES technique is the approach following the direction of lymph drainage, only half of the studies reported the anatomical distribution of the sentinel lymph nodes identified. The most frequent site was the obturator area, followed by the external iliac area, while the most infrequent site was the presacral area. This observation is similar to the laparoscopic approach, with a report of 38–70% for the external iliac area and 13–26% for the obturator area [30,33,34]. However, the obturator area is more favoured than the external iliac area by the V-NOTES approach, especially with a retroperitoneal technique, highlighting the better accessibility from a caudal to a cranial direction [16]. The most commonly used tracer was indocyanine green (ICG), consistent with broader literature supporting its superior lymphatic mapping capabilities [35]. Emerging tracer combinations, such as ICG with carbon nanoparticles, warrant further study.

Complication rates were low, with only 3.9% of patients experiencing intraoperative adverse events. Vascular injuries with haemorrhage and bladder injuries were the most frequently encountered issues. In a large retrospective study by Hou on V-NOTES for gynaecological pathologies, the reported complication rate was similar, with 4.4% [36]. This rate is comparable to the reported complication rate for the other minimally invasive techniques, such as laparoscopy with 5.6% or robotic surgery with 3.6% [37,38]. No difference in complication rates compared with classical laparoscopy is reported, also by a recent Cochrane review [39]. In Hou’s study, the most frequent complication besides haemorrhage was rectal injury, which occurred exclusively in patients with deep endometriosis. This observation corroborates the recommendation of the 2021 V-NOTES consensus to refrain from performing this technique in the presence of deep endometriosis, underscoring the significance of case selection [25]. Also, these complications highlight the learning curve associated with V-NOTES, especially in lymphatic mapping, and emphasise the need for proper training during the initial adoption phase.

This review is limited by the heterogeneity and mostly retrospective nature of the included studies. Only three were comparative cohort studies, which restricts our ability to draw definitive conclusions about the comparative efficacy or safety of V-NOTES relative to other surgical approaches. Moreover, the inclusion of multiple small case series may introduce selection bias, as these often report the most favourable outcomes during the learning phase.

Despite these limitations, the aggregated evidence suggests that V-NOTES represents a feasible and effective alternative for SLN staging in endometrial cancer. As surgical expertise grows and more robust comparative data become available, V-NOTES may assume a more prominent role in the minimally invasive staging alternatives.

## 5. Conclusions

The findings of this review suggest that V-NOTES is a promising technique for sentinel lymph node (SLN) staging in endometrial cancer, offering high detection rates, low complication rates, and favourable perioperative outcomes. While current evidence, drawn from a mix of case reports, case series, and a few observational studies, supports the safety and efficacy of V-NOTES, the limited number and heterogeneity of available studies highlight the need for larger, prospective, and comparative trials. Standardisation of technique, broader reporting of long-term oncologic outcomes, and further exploration of optimal patient selection criteria will be essential in validating the role of V-NOTES in routine clinical practice.

## Figures and Tables

**Table 1:** Characteristics of the population included.

Author	Year	Design	Patients	Mean Age	Mean BMI (kg/m^2^)	>25 kg/m^2^ (%)	Mean Parity	Prior Surgery (%)
Baekeland [11]	2024	Case series	64	69.5	26	34	-	38
Comba [12]	2024	Cohort	19	59.4	29	100	2	-
Comba [13]	2021	Case report	1	46	27.4	100	2	100
Deng [14]	2023	Cohort	57	51.46	26.25	78.95	2	31.58
Huber [15]	2022	Case series	7	68	26.4	57	2.2	57
Huber [16]	2024	Case series	34	68	27.3	-	-	58.8
Lee [17]	2022	Case series	15	52.8	27.8	26	1.8	26
Matak [18]	2024	Case series	4	67	28.45	100	2	25
Mathey [19]	2022	Case report	1	64	-	-	-	-
Simsek [20]	2024	Case series	24	56.5	31	-	-	67
Tantitamit [21]	2018	Case series	4	60.3	25.6	75	3.2	50
Wang [22]	2021	Cohort	23	53	24.2	-	2	34

**Table 2:** Pre-operative histological and imaging characteristics.

Author	Year	Tumour Histology	Tumour Grading	Imaging (US/MRI)	Staging
Baekeland [11]	2024	All types and Complex Atypical Hyperplasia	1–3	No	I-II
Comba [12]	2024	-	-	Yes	-
Comba [13]	2021	Endometrioid	2	Yes	I
Deng [14]	2023	Endometrioid	-	Yes	I
Huber [15]	2022	Endometrioid and Complex Atypical Hyperplasia	1–2	Yes	I
Huber [16]	2024	Endometrioid and Complex Atypical Hyperplasia	-	Yes	I
Lee [17]	2022	Endometrioid	1–2	Yes	I
Matak [18]	2024	Endometrioid	1	No	I
Mathey [19]	2022	Endometrioid	1	Yes	I
Simsek [20]	2024	-	-	Yes	-
Tantitamit [21]	2018	-	-	No	I
Wang [22]	2021	Endometrioid	1–2	Yes	I

**Table 3:** Surgical outcomes.

Author	Year	SLN Approach	Tracer	Mean op. Time	Estimated Blood Loss (mL)	Pre-op-Post-op HB (g/dL)
Baekeland [11]	2024	Retroperitoneal	ICG	126	80	−1
Comba [12]	2024	Retroperitoneal	ICG	208.4	76.3	−1.5
Comba [13]	2021	Retroperitoneal	ICG	180	20	-
Deng [14]	2023	Transperitoneal	ICG, CNP	132.35	75	-
Huber [15]	2022	Retroperitoneal	ICG	113	20	-
Huber [16]	2024	Retroperitoneal	ICG	-	-	-
Lee [17]	2022	Transperitoneal	ICG	231.4	122	−1.44
Matak [18]	2024	Retroperitoneal	ICG	169	-	−1.9
Mathey [19]	2022	Retroperitoneal	ICG	113	100	-
Simsek [20]	2024	Retroperitoneal	ICG	125	70	-
Tantitamit [21]	2018	Transperitoneal	ICG	182.7	67	−0.57
Wang [22]	2021	Transperitoneal	CNP	132.3	90	−1

**Table 4:** SLN mapping outcomes.

Author	Year	Succes Rate (%)	Total LN	Mean Right LN	Mean Left LN	Overall DR (%)	Bilateral DR (%)	Unilateral DR (%)
Baekeland [11]	2024	98.5	3	-	-	100	97	3
Comba [12]	2024	94.8	-	-	-	94.8	-	-
Comba [13]	2021	100	-	-	-	100	-	-
Deng [14]	2023	81.5	-	-	-	94.7	82.4	12.3
Huber [15]	2022	100	3.1	1.2	1.8	100	100	-
Huber [16]	2024	91.2	3	1	1	97.1	91.2	5.9
Lee [17]	2022	100	5.3	2.3	1.9	100	-	-
Matak [18]	2024	100	12.5	5.7	6.2	100	-	-
Mathey [19]	2022	100	3	1	2	100	100	-
Simsek [20]	2024	96	2	1	1	96	91	4
Tantitamit [21]	2018	100	8.5	5	2.5	100	100	-
Wang [22]	2021	95.7	5	-	-	95.7	87	8.7

**Table 5:** Characteristics of the retroperitoneal vs. transperitoneal approach.

Characteristic	Retroperitoneal V-NOTES	Transperitoneal V-NOTES
Access Route	Via retroperitoneal space, avoiding the peritoneal cavity	Through the peritoneal cavity from the vaginal canal
Peritoneum Penetration	Not penetrated	Penetrated to access abdominal cavity
Visualization	Limited, more challenging anatomy orientation	Excellent visualization of intra-abdominal structures
Risk of Intraperitoneal Injury	Lower	Higher (bowel, bladder, vessels)
Adhesion Risk	Reduced	Possible if adhesions present
Suitable For	Select procedures (e.g., lymphadenectomy, small masses)	Broader range of gynecologic surgeries (e.g., hysterectomy)
Learning Curve	Steeper due to complex anatomy	Less steep, similar to standard laparoscopy
CO_2_ Insufflation	Confined to retroperitoneum	Fills the peritoneal cavity
Postoperative Recovery	Potentially faster and less pain	Slightly more discomfort due to peritoneal irritation
Preferred When	Avoiding peritoneal contamination or minimizing invasiveness	When broader access is needed or for complex procedures

## Abbreviations

The following abbreviations are used in this manuscript:

V-NOTES	Vaginal Natural Orifice Transluminal Endoscopic Surgery
SLN	Sentinel Lymph Node
ICG	Indocyanine green
